# Thymol Mitigates Oxidative Stress-Induced Ovarian Aging and Restores Steroidogenesis via the JAK1–STAT3 Pathway

**DOI:** 10.3390/cimb47110910

**Published:** 2025-11-02

**Authors:** Junjie Deng, Chen Luo, Chen Xie, Heng Duan

**Affiliations:** 1 College of Traditional Chinese Medicine, Chongqing Medical University, Chongqing 400016, China; 2 College of Chinese and Modern Medicine, Chongqing University of Chinese Medicine, Chongqing 402760, China

**Keywords:** thymol, premature ovarian failure, oxidative stress, cellular senescence, JAK1–STAT3 pathway, steroidogenesis

## Abstract

Premature ovarian failure (POF) is characterized by oxidative stress, cellular senescence, and impaired steroidogenesis, yet current therapies remain limited in effectiveness. Thymol, a natural monoterpene, exhibits antioxidant and anti-inflammatory properties. Network pharmacology and molecular docking identified multiple potential targets, notably the Janus kinase 1 (JAK1)-signal transducer and activator of transcription 3 (STAT3) pathway. In tert-butyl hydroperoxide (t-BHP)-induced human granulosa-like tumor cells (*n* = 3), 40 μg/mL thymol increased cell viability by approximately 45%, restored superoxide dismutase, catalase, and glutathione peroxidase activities to nearly twice those of the model group, and reduced reactive oxygen species accumulation by about 35% (*p* < 0.05). It also decreased senescence markers p53, p21, and p16 by 40–60% and inhibited JAK1–STAT3 phosphorylation (*n* = 3, *p* < 0.05). In aged pregnant mice (*n* = 4 per group), thymol increased viable fetus numbers by about 40%, elevated serum estradiol and progesterone levels to 1.6–1.8-fold of aged controls, and downregulated ovarian aging markers (*p* < 0.05). Collectively, these findings indicate that thymol mitigates oxidative stress-induced ovarian aging by modulating JAK1–STAT3 signaling and restoring steroidogenic function, supporting its potential as a natural candidate for delaying ovarian senescence.

## 1. Introduction

Female infertility has become an increasing global health concern, affecting approximately 10–15% of couples of reproductive age [1,2]. Premature ovarian failure (POF) and age-related ovarian decline are among the leading causes of female infertility. POF is defined as the loss of ovarian activity before the age of 40 and is characterized by amenorrhea, hypoestrogenism, and elevated gonadotropins [3]. Similarly, ovarian aging occurs naturally with advancing age but is accelerated by genetic, environmental, and metabolic factors, resulting in diminished ovarian reserve and poor reproductive outcomes [4]. Despite progress in assisted reproductive technologies and hormone replacement therapy, effective interventions to preserve or restore ovarian function remain limited. Therefore, it is crucial to understand the molecular mechanisms underlying ovarian aging and to explore new therapeutic candidates [5].

Granulosa cells play a pivotal role in maintaining ovarian function by supporting oocyte growth, regulating follicular development, and producing steroid hormones such as estradiol [6]. However, granulosa cells are highly susceptible to oxidative stress, which has been recognized as a central driver of ovarian aging and POF [7]. Excessive accumulation of reactive oxygen species (ROS) damages DNA, proteins, and lipids. This damage triggers mitochondrial dysfunction and activates senescence pathways. Key regulators such as p53, p21, and p16 enforce cell cycle arrest and senescence, ultimately impairing follicular maturation and reducing the ovarian reserve [8]. In parallel, oxidative stress disrupts steroidogenesis by downregulating enzymes including aromatase (CYP19A1), steroidogenic acute regulatory protein (StAR), and follicle-stimulating hormone receptor (FSHR), resulting in decreased estradiol synthesis and impaired fertility [9]. Increasing evidence also implicates the Janus kinase 1 (JAK1)–signal transducer and activator of transcription 3 (STAT3) signaling pathway in mediating oxidative stress responses, inflammation, and cellular senescence, making it a potential target in ovarian aging research [10].

In recent years, plants have emerged as cost-effective and safe sources of natural antioxidants, with growing research examining their role in alleviating ovarian aging. Natural antioxidants such as resveratrol, a polyphenol found in grapes and red wine, have demonstrated anti-aging effects; oral administration in aged mice delays reproductive decline and preserves fertility [11]. Melatonin, an endogenous antioxidant and anti-inflammatory hormone, is present at high levels in ovarian follicular fluid. Beyond regulating circadian rhythms, it enhances expression of the steroidogenic protein StAR in granulosa cells, promoting progesterone production and luteal function [12]. Additionally, flavonoids and polyphenols such as quercetin and curcumin protect ovarian cells by mitigating oxidative stress and apoptosis [13]. Collectively, these findings suggest that natural antioxidants can enhance the ovarian microenvironment, delaying cellular aging and supporting steroid hormone synthesis. However, most existing studies have focused on polyphenols and flavonoids, while the potential role of volatile monoterpenoids such as thymol in ovarian protection remains largely unexplored.

Thymol, a dietary monoterpenoid phenol found abundantly in plants such as *Thymus vulgaris*, *Ocimum gratissimum*, and *Thymus ciliatus*, exhibits antimicrobial, anti-inflammatory, and antioxidant activities [14]. Combined with Constitutive Androstane Receptor, it significantly reduces oxidative stress and protects ovarian reserve, highlighting its potential for ovarian preservation [15]. However, the molecular mechanisms through which thymol exerts its protective effects on the ovary remain unclear. Our previous metabolomics analysis of the characteristic Chinese citrus variety *Citrus reticulata* ‘Chachi’, which identified β-caryophyllene, thymol, and γ-terpinene as candidate antioxidants, suggesting that thymol may exhibit distinctive bioactivity among volatile compounds [16]. Building upon these findings, our study integrates network pharmacology and experimental validation to investigate the protective effects of thymol against oxidative stress-induced ovarian aging and to elucidate its underlying mechanisms.

## 2. Materials and Methods

### 2.1. Network Pharmacology and Molecular Docking

For network pharmacology analysis, the chemical structures of γ-terpinene, β-caryophyllene, and thymol were obtained from the PubChem database based on their SMILES IDs. Their corresponding targets were predicted using the SwissTargetPrediction database and further filtered in the STRING database with a confidence score threshold of ≥0.8. Potential disease-related targets for POF were retrieved from the GeneCards, OMIM, TTD, and DisGeNET databases using the keyword “Premature Ovarian Failure.” Only targets with a predicted probability score of ≥0.1 were considered for further analysis, in order to ensure a reliable confidence level and minimize false-positive predictions. Venn analysis was performed using the R package “Venn” (version 4.0.2) to identify overlapping targets between the POF- and compound-related datasets. These overlapping targets were prioritized for further analysis, as they represent the potential molecular interfaces through which the compounds may exert therapeutic effects on POF. The common targets were used to construct a compound–target network in Cytoscape (version 3.8.0). Protein–protein interactions (PPIs) were analyzed in the STRING database (confidence score ≥ 0.9) and visualized in Cytoscape. Topological parameters (degree, betweenness, eigenvector, and closeness centrality) were calculated using the CytoNCA plugin. Gene Ontology (GO) and Kyoto Encyclopedia of Genes and Genomes (KEGG) pathway enrichment analyses were performed using ClusterProfiler, DOSE, and Enrichplot (version 4.0.2), with *p* ≤ 0.05 considered statistically significant.

For molecular docking, the 3D structure of thymol was obtained from PubChem, and protein structures were obtained from RCSB PDB, with X-ray resolutions better than 2.5 Å to ensure structural reliability for docking. First, thymol was docked with all core targets identified from the network pharmacology analysis to evaluate binding affinities. The binding energies for thymol across all targets ranged from −4.8 to −7.6 kcal/mol, with lower values indicating more stable interactions. Complexes with binding energies below −5.0 kcal/mol were considered to have strong binding potential. Among all docked proteins, JAK1, SLC6A3, and ACHE exhibited the lowest binding energies and were therefore selected for visualization in PyMOL (v4.5.0). Proteins were prepared using PyMOL to remove water and solvent molecules and complexes were visualized with PyMOL.

### 2.2. Cell Culture and Treatments

#### 2.2.1. Human Granulosa-like Tumor Cell Line, KGN Culture

The human granulosa-like tumor cell line KGN (BNCC337610, BeNa Culture Collection, Beijing, China) was used in this study. Cells were maintained in DMEM/Ham’s F12 medium (G4611-500ML, Servicebio, Wuhan, China) containing 10% fetal bovine serum (3023A, Umedium, GeZhe Biotechnology, Hefei, China) and 1% penicillin-streptomycin (G4016-100ML, Servicebio, Wuhan, China). Cultures were incubated at 37 °C in a humidified atmosphere with 5% CO_2_. When cell confluence reached about 80%, they were passaged for subsequent experiments.

#### 2.2.2. Cell Viability Assay

Various concentrations of thymol were selected based on its solubility (approximately 1 mg/mL) in water [17] to evaluate its effects on KGN cell viability. KGN cells (1 × 10^4^/well) were seeded in 96-well plates and treated for 24 h with thymol (0, 5, 10, 20, 40, 80, 160, 320 and 640 μg/mL, HY-N6810, MedChemExpress, Monmouth Junction, NJ, USA) or tert-Butyl hydroperoxide (t-BHP) (0, 25, 50, 100, 200 and 400 μM, St. Louis, Sigma, MO, USA). To induce oxidative stress, cells were exposed to t-BHP (100 μM, 24 h) with or without thymol (0, 5, 10, 20 and 40 μg/mL). Viability was assessed with Cell Counting Kit-8 (G1613-1 ML, Servicebio, Wuhan, China). After treatment, 10 μL of CCK-8 solution (10% of the total culture volume) was added to each well and incubated for 2 h at 37 °C before measuring absorbance at 450 nm.

#### 2.2.3. Measurement of Oxidative Stress Markers

Superoxide dismutase (SOD, S0101S, Beyotime, Shanghai, China), catalase (CAT, S0051, Beyotime, Shanghai, China), and glutathione peroxidase (GPx, S0056, Beyotime, Shanghai, China) activities, and malondialdehyde (MDA, S0131S, Beyotime, Shanghai, China) levels were measured using commercial kits according to the manufacturer’s instructions.

#### 2.2.4. ROS Staining

Intracellular ROS levels were determined using the fluorescent probe 2′,7′-dichlorodihydrofluorescein diacetate (DCFH-DA, G1706-100T, Servicebio, Wuhan, China). And were observed and photographed under a fluorescence microscope (excitation 488 nm, emission 525 nm). Quantitative analysis was performed using ImageJ software (version 1.54p).

#### 2.2.5. Senescence-Associated β-Galactosidase (SA-β-Gal) Staining

To assess the degree of KGN cells senescence, cells were stained using a cellular senescence β-galactosidase staining kit (C0602, Beyotime, Shanghai, China). The staining was observed under a microscope, and ImageJ software was used to calculate the area of positive staining.

#### 2.2.6. RNA Extraction and Quantitative Real-Time Polymerase Chain Reaction (RT-qPCR) Analysis

A total of 1 × 10^5^ cells were used for RNA extraction. Total RNA was isolated using the SteadyPure Mag RNA Kit (AG21205, Accurate Biology, Changsha, China) according to the manufacturer’s instructions. RNA concentration and purity were measured with a NanoDrop 2000 spectrophotometer (Thermo Fisher Scientific, Wilmington, DE, USA). Complementary DNA (cDNA) was synthesized using the Evo M-MLV Plus cDNA Synthesis Kit (AG11615, Accurate Biology, Changsha, China). RT-qPCR awas performed in triplicate using the SYBR Green SupTaq HS kit (AG11762, Accurate Biology, Changsha, China) according to the manufacturer’s instructions. Gene expression levels were normalized to β-actin. The primer sequences used are shown in Table 1.

### 2.3. Animal Experiment

#### 2.3.1. Drug Preparation

The dosing regimen of thymol was determined based on previous reports [18,19], and 30 mg/kg was selected as the high dose for preliminary evaluation of its protective effect. Briefly, 6 mg of thymol was dissolved in 1 mL of purified water to prepare a 6 mg/mL solution. Solutions of medium (3 mg/mL) and low (1.5 mg/mL) concentrations were prepared in the same manner.

Progesterone (HY-N0437, MedChemExpress, Monmouth Junction, NJ, USA) was initially dissolved in Dimethyl sulfoxide (DMSO). After complete dissolution, corn oil (HY-Y1888, MedChemExpress, Monmouth Junction, NJ, USA) was added at a ratio of 1:9 (*v*/*v*, DMSO–corn oil) to obtain a 6 mg/mL progesterone solution, which served as the positive control for the POF mice. The final DMSO concentration in the working solution was therefore 10% (*v*/*v*).

#### 2.3.2. Animal Handling

Three months old female C57BL/6 mice (25 ± 3 g), twelve months old female C57BL/6 mice (30 ± 3 g) and twelve-week-old male C57BL/6 mice (20 ± 3 g) were purchased from Hunan SJA Laboratory Animal Co., Ltd., Changsha, China. All mice were housed in specific pathogen-free facility at Chongqing Medical University under a 12 h/12 h light/dark cycle, at 22–25 °C and 25 kPa atmospheric pressure. Food and water were available ad libitum throughout the experiment. All animal experiments were conducted in accordance with the approval of the Ethics Committee of Chongqing Medicine University Ethics (Ethics Approval No: IACUC-CQMU-2025-09062).

After a week of adaptive feeding, female and male mice were housed together at a ratio of 2:1 at 5 p.m. At 8 a.m. the following day, vaginal plugs were examined; female mice with plugs were confirmed as successfully mated, marking Gestation Day 0.5 (D0.5) of pregnancy. The Young Pregnant group (YP) comprised young mice that had formed vaginal plugs. While other old female mice were randomly divided into five groups (*n* = 4/group) using a computer-generated random number list: Aged pregnant group (AP), thymol low dose group (thymol-L), thymol medium dose group (thymol-M) and thymol high dose group (thymol-H). The Progesterone (P) group received a daily intramuscular injection of 0.1 mL 2 mg/mL P. Thymol-H group received 30 mg/kg body weight thymol during the same period. Thymol-M and thymol-L groups received thymol at doses of 15 mg/kg and 7.5 mg/kg, respectively. The other two groups received 0.1 mL of pure water during the same period. Serum samples were obtained via orbital sinus blood collection on D0.5, 7.5, and 13.5 for the assessment of pregnancy-associated hormones. On D13.5, the mice were euthanized using excess isoflurane, and ovaries were collected. Part of the ovarian tissue was fixed in 4% paraformaldehyde (G1101-3ml, Servicebio, Wuhan, China), while the remaining tissue was stored at −80 °C for further analysis. Mice with normal health status, normal estrous cycles, and body weights within the specified ranges were included in the study. Animals exhibiting irregular estrous cycles, abnormal behavior, illness, or body weight deviations exceeding ±20% were excluded from analysis.

#### 2.3.3. Hematoxylin-Eosin (HE) Staining Analysis

To analyze ovarian morphology, D13.5 ovaries collected from the six groups were fixed in 4% paraformaldehyde for 12–16 h. Following fixation, tissue samples were dehydrated, embedded in paraffin, and sectioned continuously at a thickness of 5 μm and stained with HE. Ovarian sections were imaged using a Leica TCS SP8 confocal laser scanning microscope (Leica Microsystems, Wetzlar, Germany) in panoramic scanning mode to capture the entire ovarian morphology.

#### 2.3.4. Quantification of Mature Follicles, Atretic Follicles, and Corpora Lutea in the Ovary

In brief, every sixth ovarian section was examined under a light microscope. Morphological criteria were applied to identify different follicular structures: mature follicles (well-developed antral cavity, intact oocyte, and continuous granulosa cell layers), atretic follicles (granulosa cell vacuolization, nuclear fragmentation, or degenerative oocyte), and corpora lutea (compact luteal cell clusters with preserved morphology). Follicle counts were independently performed by two blinded observers. When discrepancies exceeded 10%, a third observer re-evaluated the slides, and the median value was adopted. For each animal, the total numbers of mature follicles, atretic follicles, and corpora lutea per ovary were recorded. Data were presented as mean ± SD, and statistical comparisons among groups were conducted using one-way ANOVA followed by Tukey’s post hoc test (or a non-parametric test when appropriate). A *p* < 0.05 was considered statistically significant.

### 2.4. Enzyme-Linked Immunosorbent Assay (ELISA)

The levels of P (GEM0015-48T, Servicebio, Wuhan, China), mouse estradiol (E2) (CEA461Ge 96T, Cloud-Clone Corp., Houston, TX, USA) and Human Estradiol (XY-1899, Shanghai Xinyu Corp., Shanghai, China) were measured using ELISA kits according to the manufacturer’s instructions. The detection ranges were 0–50 ng/mL for P, 0.02–4 ng/mL for mouse E2, and 0–400 ng/mL for human E2. The intra- and inter-assay coefficients of variation were <10% and <12%, respectively.

### 2.5. Western Blot

Total proteins were extracted from ovarian tissue or KGN cells using RIPA lysis buffer combined with the protease inhibitor phenylmethylsulfonyl fluoride (G2002-30ml, G2008-1ml, Servicebio, Wuhan, China) and phosphatase inhibitor (G2007-1ML, Servicebio, Wuhan, China). The lysate was collected by centrifugation at 12,000 rpm for 15 min at 4 °C. Total protein concentration was determined using a bicinchoninic acid protein assay (P0010S, Beyotime, Shanghai, China), and samples were then diluted to 4 μg/mL. Protein samples were separated using SWE rapid high-resolution electrophoresis buffer (G2081-1L, Servicebio, Wuhan, China) and transferred to a 0.45 μm polyvinylidene difluoride membrane (G6047-0.45, Servicebio, Wuhan, China). The membranes were sealed with 5% skim milk dissolved in Tris-buffered saline with Tween-20. Subsequently, all membranes were incubated with antibodies at 4 °C for 12 h: p53 (1:1000, 345567, Zenbio, Chengdu, China), P21 (1:1000, 381102, Zenbio, Chengdu, China), CDKN2A/p16INK4a (1:1000, R23897, Zenbio, Chengdu, China), STAT3 (1:2000, STAT3, Zenbio, Chengdu, China), Phospho-STAT3 (Tyr705) (1:1000, 340799, Zenbio, Chengdu, China), JAK1 (1:1000, 310108, Zenbio, Chengdu, China), Phospho-JAK1 (Tyr1034/Tyr1035) (1:2000, R30275, Huabio, Hangzhou, China). The next day, membranes were incubated with secondary antibodies (goat anti-rabbit IgG, GB23303, Servicebio, Wuhan, China) at 25 °C for 1 h. Subsequently, BeyoECL plus (P0018S, Beyotime, Shanghai, China) was applied to the bands, which were visualized using the C300 system. ImageJ was used to analyze the grayscale values.

### 2.6. Statistical Analysis

All experiments were independently repeated at least three times. For cell-based assays (e.g., viability, oxidative stress markers, ROS, SA-β-Gal staining, and qPCR), three biological replicates were performed, and each measurement was repeated in triplicate (technical replicates). For animal experiments, four mice were included per group (*n* = 4). Randomization was applied to assign animals into experimental groups, and investigators performing histological and follicle counting analyses were blinded to group allocation to minimize bias. Data were expressed as mean ± standard deviation (SD). For comparisons between two groups, an unpaired two-tailed Student’s *t*-test was used. For comparisons among multiple groups, one-way analysis of variance (ANOVA) followed by Tukey’s post hoc test was performed to account for multiple comparisons. When data did not meet the assumptions of normality or equal variance, the Kruskal–Wallis test followed by Dunn’s post hoc test was used. A *p* value < 0.05 was considered statistically significant. All analyses were conducted using GraphPad Prism Software (version 9.0).

## 3. Results

### 3.1. Network Pharmacology Analysis Reveals a Potential Role of Thymol in Modulating POF

**Figure 1:**
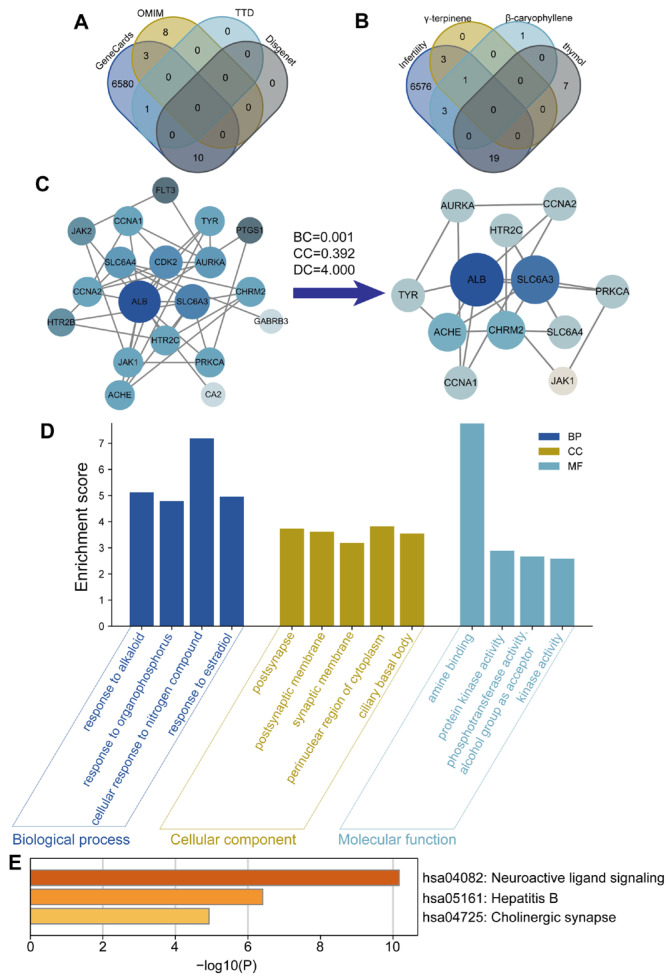
Network pharmacology analysis of thymol in primary ovarian failure. (**A**) Venn diagram showing POF-related targets identified from GeneCards, OMIM, TTD, and DisGeNET databases. (**B**) Venn diagram depicting overlap between POF targets and potential targets of thymol, γ-terpinene, and β-caryophyllene. (**C**) Interaction network of shared targets between thymol and POF, highlighting 12 core targets selected using Cytoscape. The darker color and larger circle size indicate nodes with higher degree values. (**D**) Gene Ontology enrichment analysis of the 12 core targets. (**E**) Kyoto Encyclopedia of Genes and Genomes pathway enrichment analysis of the 12 core targets.

To predict the possible targets of γ-terpinene, β-caryophyllene and thymol, we utilized the PubChem, Swiss Target Prediction, and STRING databases, identifying 4 potential targets for γ-terpinene, 5 for β-Caryophyllene, and 26 for thymol (Figure 1A). Because γ-terpinene and β-caryophyllene yielded too few predicted targets to enable a thorough investigation of their therapeutic mechanisms, we selected thymol for subsequent experimental studies and validation. Then, using the GeneCards, TTD, DisGeNET and OMIM databases, 6594, 1, 10 and 11 potential targets for POF were identified, respectively. After eliminating duplicate targets, the final number of potential targets related to POF was 6602 (Figure 1B), and Venn diagram analysis revealed 19 key overlapping targets. PPI network analysis and the STRING database (with a medium confidence level of 0.900) were used to detect interactions and correlated functions among the 19 predicted targets, and PPI network topology was analyzed using the CytoNCA plugin. Among the 19 predicted targets, 12 were identified as core therapeutic targets (Figure 1C, Table 2). Using GO functional annotation, 8 terms were enriched in cellular component (CC) category, 5 in molecular function (MF) category, and 44 in biological process (BP) category. The BP terms were mainly related to cellular response to nitrogen compounds, response to cocaine, and response to alkaloids. Those in the CC category were mainly involved in perinuclear region of cytoplasm and ciliary basal body, and those in the MF category were mainly related to amine binding and protein kinase activity (Figure 1D). KEGG enrichment analysis identified 3 signaling pathways, including Neuroactive ligand–receptor interaction, Hepatitis B and Cholinergic synapse (Figure 1E). These findings suggest that thymol may exert anti-inflammatory effects by regulating key signaling pathways, including the STAT-JAK signaling pathways.

### 3.2. Molecular Docking of Thymol

**Figure 2:**
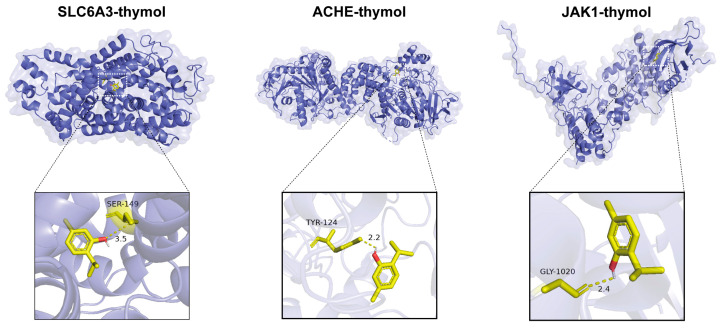
PyMOL visualization of thymol docked with the top three targets ranked by binding energy: SLC6A3, ACHE, and JAK1.

To further explore the thymol–core target interactions, we conducted molecular docking between thymol and the 12 POF-related core targets. Among these targets, Solute Carrier Family 6 Member 3 (SLC6A3), Acetylcholinesterase (ACHE), and JAK1 showed the strongest binding affinities to thymol (Table 3). The detailed 3D docking and interaction analyses revealed that thymol contained favorable binding sites for SLC6A3, ACHE, and JAK1, supporting their roles as key active compounds in treating POF (Figure 2).

### 3.3. Protective Effect of Thymol on t-BHP-Treated KGN Cells

**Figure 3:**
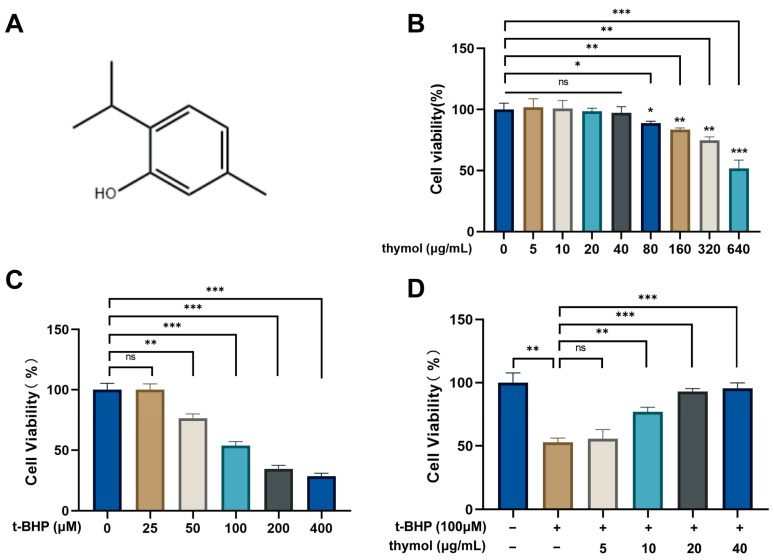
Effects of thymol on KGN cell viability. (**A**) Chemical structure of thymol. (**B**) CCK-8 assay showing KGN cell viability after treatment with different concentrations of thymol. (**C**) CCK-8 assay showing KGN cell viability after treatment with different concentrations of t-BHP. (**D**) CCK-8 assay showing the protective effects of different concentrations of thymol on t-BHP-induced KGN cells. ns, not significant; * *p* < 0.05, ** *p* < 0.01, *** *p* < 0.001.

To evaluate the potential effect of thymol (its chemical structure shown in Figure 3A) on KGN cells, we first assessed its cytotoxicity. Cell viability was measured using CCK-8 assay. As shown in Figure 3B, thymol at concentrations below 80 μg/mL for 24 h showed no significant effect on cell viability compared with control group. t-BHP is commonly used to establish an oxidative stress model in KGN cells. As the concentration of t-BHP increased, cell viability gradually decreased. The IC50 value of t-BHP was determined to be 100 µM after 24 h of treatment (Figure 3C). Therefore, 100 µM t-BHP was used in the subsequent experiments. To examine whether thymol could protect cells from t-BHP-induced injury. KGN cells were treated with different concentrations of thymol together with 100 µM t-BHP for 24 h. As shown in Figure 3D, thymol at concentrations of 10, 20, 40 μg/mL significantly improved cell viability compared with the t-BHP-only group. These findings indicate that thymol alleviates t-BHP-induced cytotoxicity in KGN cells.

### 3.4. Thymol Attenuates t-BHP-Induced Oxidative Stress and Senescence in KGN Cells

**Figure 4:**
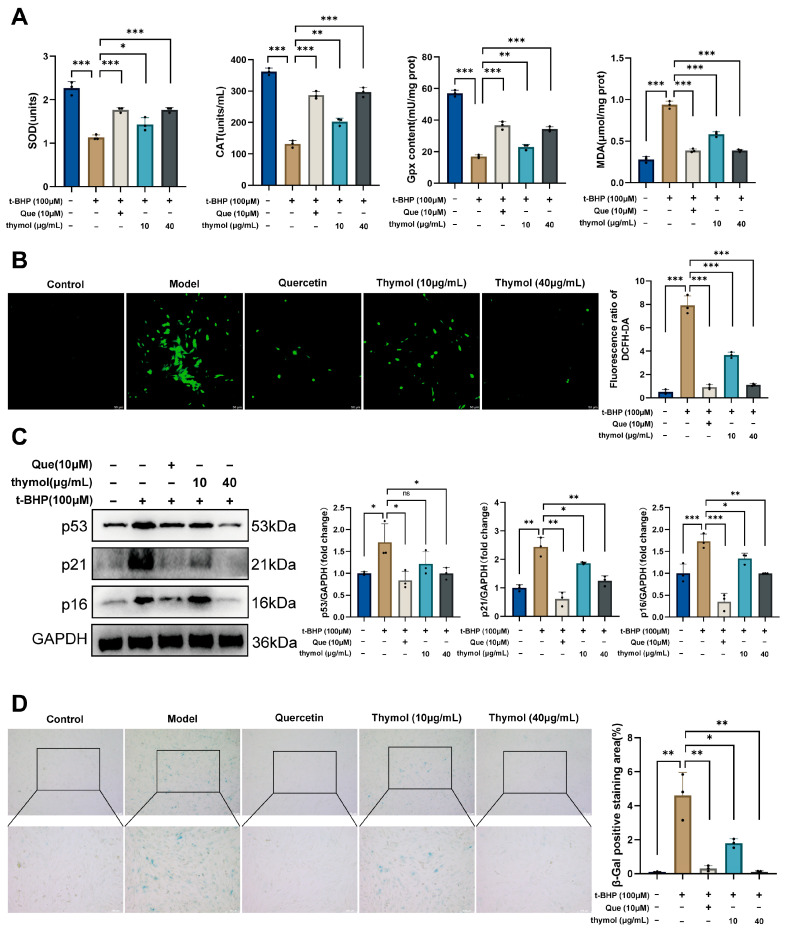
Effects of thymol on oxidative stress and senescence in KGN cells. (**A**) Levels of SOD, CAT, GPx, and MDA in KGN cells of different treatment groups. (**B**) ROS fluorescence in KGN cells of different treatment groups. (**C**) Protein expression of p53, p21, and p16 in KGN cells of different treatment groups. (**D**) β-Gal staining in KGN cells of different treatment groups. Data are presented as mean ± SD; ns, not significant; * *p* < 0.05, ** *p* < 0.01, *** *p* < 0.001.

To assess thymol’s antioxidant effects, we measured oxidative stress-related markers in KGN cells. Treatment with t-BHP significantly reduced SOD, CAT, and GPx activities while increasing MDA and ROS levels, confirming oxidative injury. Both quercetin (positive control) and thymol treatment restored antioxidant enzyme activities and reduced MDA and ROS accumulation, with the high thymol dose showing a dose-dependent effect (Figure 4A,B). We next examined cellular senescence. t-BHP exposure upregulated p53, p21, and p16 expression, whereas quercetin and high-dose thymol significantly attenuated these increases; low-dose thymol mainly reduced p21 expression (Figure 4C). Consistently, SA-β-Gal staining revealed that thymol and quercetin both reduced SA-β-Gal-positive staining area (Figure 4D). Together, these results demonstrate that thymol mitigates t-BHP-induced oxidative stress and cellular senescence.

### 3.5. Thymol Modulates JAK1–STAT3 Pathway, Restores Steroidogenic Gene Expression and Estradiol Synthesis in KGN Cells

**Figure 5:**
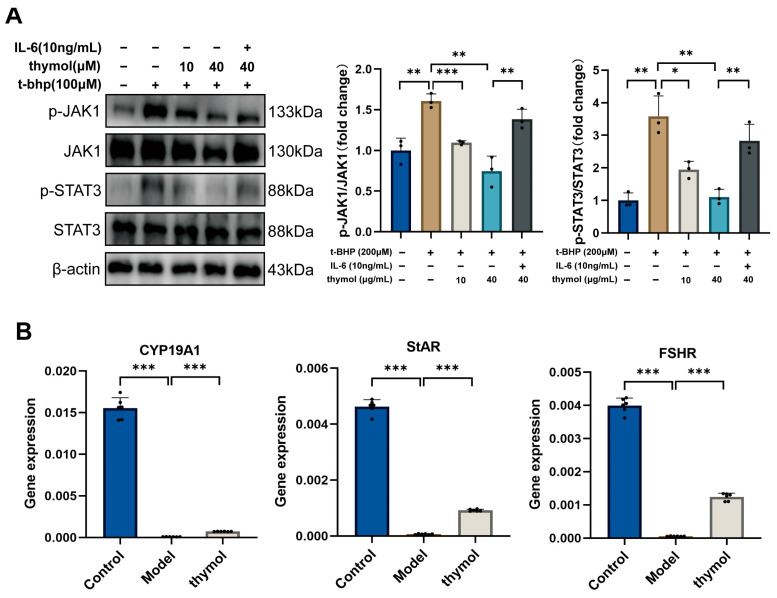
Thymol regulated JAK1–STAT3 signaling and upregulated steroidogenic genes, supporting its role in improving ovarian function. (**A**) Protein expression of p-JAK1, JAK1, p-STAT3, and STAT3 in KGN cells from the control, aging model, thymol low-dose, thymol high-dose, and IL-6 groups, as determined by Western blot analysis. (**B**) Relative mRNA expression levels of CYP19A1, StAR, and FSHR in KGN cells following different treatments. Data are presented as mean ± SD. * *p* < 0.05, ** *p* < 0.01, *** *p* < 0.001.

Molecular docking identified SLC6A3, ACHE, and JAK1 as top thymol-binding targets. JAK1 was prioritized because of its essential role in primordial follicle maintenance and cellular aging. WB analysis showed unchanged total JAK1 and STAT3 levels but a marked increase in p-JAK1 and p-STAT3 after t-BHP treatment. High-dose thymol reduced phosphorylation of both proteins. Interleukin-6 (IL-6) stimulation re-elevated p-JAK1 and p-STAT3 in thymol-treated cells, weakening its protective effect. These findings indicate that thymol alleviates senescence partly by inhibiting JAK1–STAT3 activation (Figure 5A). ELISA showed a significant decline in E2 levels in senescent KGN cells, which was reversed by thymol treatment (Table 4). qPCR analysis revealed that senescence suppressed CYP19A1, StAR, and FSHR expression, whereas thymol significantly upregulated all genes (Figure 5B). These results suggest that thymol supports steroidogenic function by activating key genes and restoring E2 production.

### 3.6. Thymol Improves Reproductive Outcomes and Alleviates Ovarian Aging in POF Mice

**Figure 6:**
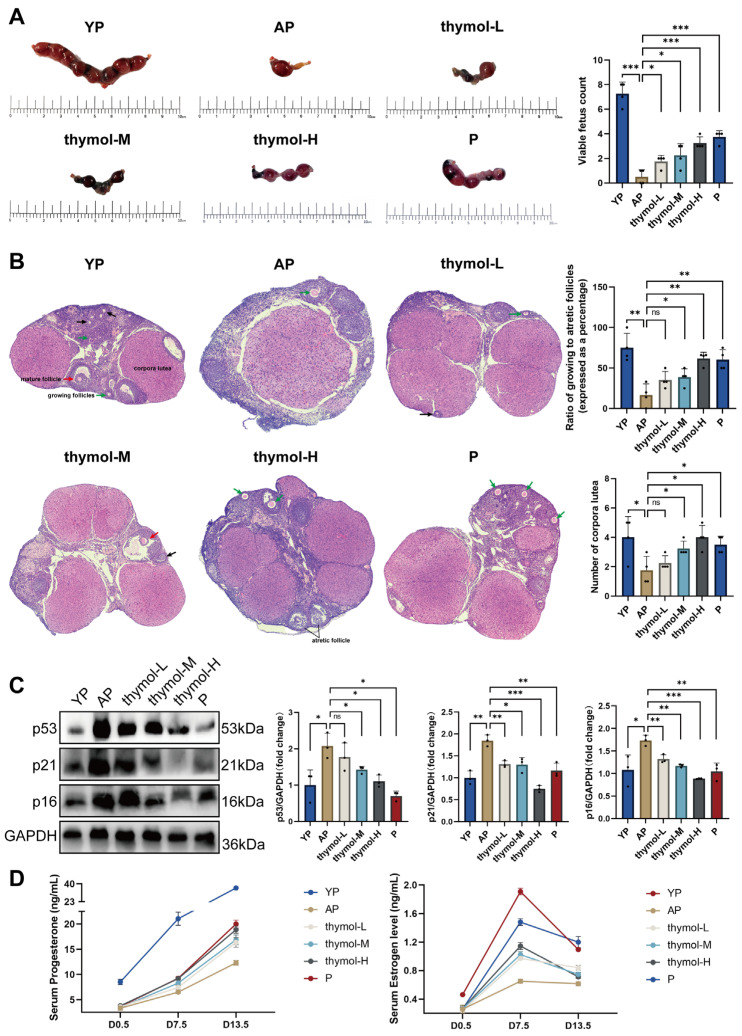
Effects of thymol on ovarian morphology and function in mice. (**A**) Representative images of uterus–fetus anatomy (with fetuses) at gestational day (**D**) 13.5 and graph of viable fetus counts (*n* = 4). (**B**) Representative ovarian HE sections of mice at D13.5; green, red, and black arrows indicate growing follicles, mature follicles, and atretic follicles, respectively. (**C**) Protein expression of p53, p21, and p16 in ovarian tissue. (**D**) Serum concentrations of progesterone and estradiol in mice at D0.5, D7.5, and D13.5 of pregnancy. Data are presented as mean ± SD; ns, not significant; * *p* < 0.05, ** *p* < 0.01, *** *p* < 0.001.

To assess in vivo effects, we established an ovarian aging mouse model and administered various concentrations of thymol or P (positive control) throughout pregnancy. On D13.5, uterine inspection revealed that YP mice carried multiple well-developed fetuses, whereas AP mice exhibited a marked reduction in fetal number, with fetuses predominantly localized to one uterine horn. Treatment with thymol at all doses, as well as P, significantly increased the number of viable fetuses (Figure 6A). Histological analysis of ovarian tissues further supported these findings: YP mice displayed abundant growing and mature follicles along with multiple corpora lutea, while AP mice showed follicular depletion and atrophy. Except for the low-dose thymol group did not significantly change corpus luteum counts or atretic follicle proportion; other groups showed increased numbers of corpora lutea and a higher proportion of developing follicles (Figure 6B). Western blot analysis further demonstrated that, compared with YP mice, AP mice showed elevated expression of ovarian aging markers (p53, p21, and p16), indicating POF. Although low-dose thymol did not significantly reduce p53 expression, it markedly decreased p21 and p16 levels. All other treatments significantly alleviated ovarian aging, suggesting that the reproductive benefits of thymol may be attributable to delayed ovarian aging (Figure 6C). Maternal hormone levels are critical throughout pregnancy. Serum P and E2 were measured at D0.5, D7.5, and D13.5, and thymol treatment significantly increased P and E2 levels in POF mice, indicating its beneficial effects on pregnancy outcomes (Figure 6D).

## 4. Discussion

Ovarian aging forms a vicious cycle where oxidative stress accelerates cellular senescence, leading to impaired steroidogenic function and reduced estradiol levels. This hormonal decline further exacerbates follicular atresia and ovarian dysfunction [20]. However, this physiological process may differ substantially between humans and mice. Both species exhibit a decline in follicle number and oocyte quality with age, but mice exhaust their ovarian reserve much earlier relative to lifespan [21,22]. Likewise, aged mice enter an estropause state without the drastic hormonal collapse of human menopause. Humans uniquely show substantial ovarian fibrosis and stromal scarring by midlife, a pathology largely absent in mice of equivalent age [23]. These interspecies differences imply that murine models, despite recapitulating many aging features, may not fully mimic the timeline or tissue microenvironment changes of human ovarian aging. Therefore, ovarian anti-aging findings in mice should be translated to humans with caution.

Notably, both network pharmacology and molecular docking analyses identified JAK1 as a potential direct binding target of thymol, implying a dual mechanism of action. Thymol may directly interact with JAK1 to inhibit its phosphorylation while simultaneously mitigating oxidative stress, which further reduces JAK1–STAT3 pathway activation. This dual regulatory mode provides a more comprehensive explanation for thymol’s antioxidative and antiapoptotic effects. Beyond the JAK1–STAT3 pathway identified in this study, thymol may exert broader protective effects against ovarian aging through multiple signaling cascades. Thymol has been reported to activate the PI3K/Akt pathway, thereby promoting cell survival and attenuating apoptosis in oxidative or inflammatory environments [24]. This effect may enhance granulosa cell viability and follicular development. Recent studies suggest that thymol may exert broader reproductive benefits beyond its antioxidant and anti-inflammatory activities. In a γ-irradiation-induced ovarian failure model, thymol significantly restored follicular structure, reduced oxidative stress, and downregulated inflammatory mediators, thereby preserving ovarian reserve and hormonal balance [15]. Similarly, in an LPS-induced ovarian injury model, thymol treatment increased serum E2 and anti-Müllerian hormone levels, improved follicular morphology, and suppressed inflammatory cytokines [25]. Furthermore, combining thymol with other natural compounds may enhance its efficacy through synergistic mechanisms. For instance, thymol exhibits complementary antioxidant properties to hesperidin, and their combination provides superior protection against oxidative injury [26]. Together, these findings indicate that thymol’s therapeutic potential extends beyond JAK1–STAT3 regulation, involving interconnected PI3K/Akt pathways and recover ovarian functions, and that rational phytochemical combinations could offer a promising strategy to counteract ovarian aging.

Resveratrol and quercetin are well-studied polyphenols with notable anti-senescence properties: resveratrol can activate sirtuin pathways to improve aging ovarian function [11], and quercetin can delay ovarian aging by reducing ovarian cell senescence [27]. Both show promise in mitigating age-related infertility, but their clinical translation is constrained by poor bioavailability and the need for high or combinatorial dosing. In contrast, melatonin and curcumin act as potent antioxidants that protect reproductive tissues: long-term melatonin treatment in mice significantly preserved ovarian reserve (increasing follicle counts, litter size, and oocyte quality) [28], while curcumin supplementation delayed ovarian aging by enhancing follicular survival, embryo development, and reducing oxidative stress [29]. Melatonin’s favorable safety profile and existing clinical use make it a feasible candidate for fertility interventions, whereas curcumin’s translational impact remains limited by low systemic bioavailability despite extensive preclinical evidence. Thymol, a less-studied monoterpenoid phenol, has shown unique efficacy in preserving fertility under stress conditions (e.g., rescuing ovarian reserve after radiotherapy in rodents) [15]. Compared to the more established compounds, thymol’s potential therapeutic window appears promising, combining antioxidant and anti-inflammatory actions with presumed low toxicity, but its overall effectiveness and safety require further validation before it can be advanced as an anti-aging or infertility therapy.

A major strength of this study is the inclusion of in vivo reproductive outcomes. While many studies on natural compounds focus on cellular or molecular endpoints, we demonstrated that thymol treatment improved progesterone and estradiol levels, increased viable fetuses, and partially restored follicular and corpus luteum numbers in aged pregnant mice. These findings directly link thymol’s molecular effects to functional fertility outcomes, enhancing its translational relevance. Similarly, other studies have shown that natural compounds can remodel the ovarian microenvironment in aged female mice, restore endocrine function, and thereby improve fertility [30]. From a clinical perspective, systematic reviews confirm that antioxidant therapy in women with diminished ovarian function significantly increases the rates of high-quality embryos and clinical pregnancy [31]. Furthermore, t-BHP-induced KGN cell model primarily represents oxidative stress rather than the full physiological process of ovarian aging, oxidative damage is a central hallmark of ovarian senescence. This model therefore provides a reproducible and controllable system to assess antioxidant responses in granulosa cells. Future studies using natural aging or genetic models will help further validate these findings.

This study has several limitations. First, the sample sizes were modest, which may limit statistical power and generalizability. Second, although our data implicate the JAK1–STAT3 pathway, the mechanistic depth is limited; the causal relationship between oxidative stress modulation and JAK1–STAT3 inhibition remains to be experimentally validated. Third, the dose–response relationship of thymol was only partially characterized, and an optimal therapeutic window has not been defined. These constraints should be addressed in future studies using larger cohorts, natural or genetic aging models, pathway perturbation experiments, and formal pharmacokinetics/toxicology analyses.

## 5. Conclusions

In summary, our findings demonstrate that thymol, a bioactive compound, exerts protective effects against ovarian aging by alleviating oxidative stress-induced granulosa cell senescence. Mechanistically, thymol inhibits JAK1–STAT3 signaling, restores antioxidant enzyme activity, and downregulates senescence markers, thereby preserving granulosa cell function. Importantly, thymol also enhances steroidogenic gene expression, increases estradiol secretion, and improves reproductive outcomes in aged mice. These results suggest that thymol may serve as a potential natural candidate for delaying ovarian aging and mitigating infertility associated with premature ovarian failure. Further studies, including pharmacokinetic evaluation and clinical investigations, are needed to confirm its translational relevance to human reproductive health.

## Figures and Tables

**Table 1:** Primer sequences used for quantitative real-time PCR analysis of target genes (CYP19A1, StAR, FSHR, and β-actin).

Gene	Sequence (5′-3′)
*CYP19A1*	F:GACGCAGGATTTCCACAGAAGAG R:ATGGTGTCAGGAGCTGCGATCA
*StAR*	F:TACGTGGCTACTCAGCATCGAC R:TCAACACCTGGCTTCAGAGGCA
*FSHR*	F:GGTTTGTCCTCACCAAGCTTCG R:GGTTGGAGAACACATCTGCCTC
*β-actin*	F:CATGTACGTTGCTATCCAGGC R:CTCCTTAATGTCACGCACGAT

**Table 2:** Characteristic parameters of core target.

Name	Clustering Coefficient (CC)	Betweenness Centrality (BC)	Degree
ALB	0.69	0.54	11
SLC6A3	0.58	0.36	7
SLC6A4	0.42	0.11	5
AURKA	0.40	0.04	5
HTR2C	0.41	0.00	4
CCNA2	0.47	0.01	4
PRKCA	0.51	0.04	4
JAK1	0.47	0.01	4
CCNA1	0.47	0.01	4
CHRM2	0.43	0.02	4
TYR	0.49	0.05	4
ACHE	0.53	0.07	4

**Table 3:** Binding energies of thymol docked with the top ten core targets.

Gene	*SLC6A3*	*ACHE*	*JAK1*	*HTR2C*	*CCNA2*	*CHRM2*	*ALB*	*TYR*	*SLC6A4*	*AURKA*	*PRKCA*	*CCNA1*
Binding energy (kcal/mol)	−7.2	−7.1	−7.0	−6.9	−6.8	−6.8	−6.7	−6.7	−6.5	−6.2	−5.7	N/A

N/A, not applicable.

**Table 4:** The comparison of E2 levels in KGN cells. ** *p* < 0.01 compared to Control group, ## *p* < 0.01 compared to Model group.

Group	Number	Estradiol (ng/mL)
Control	4	248.38 ± 8.38
Model	4	157.85 ± 11.08 **
thymol	4	201.08 ± 9.65 ^##^

## Data Availability

The datasets generated and/or analyzed during the current study are available from the corresponding author on reasonable request. The human ovarian granulosa-like tumor cell line (KGN) was purchased from the BeNa Culture Collection (BNCC337610, Beijing, China).

## Abbreviations

The following abbreviations are used in this manuscript:

POF	Premature Ovarian Failure
YP	Young Pregnant
AP	Aged Pregnant
P	Progesterone
E2	Estradiol
HE	Hematoxylin and Eosin
ELISA	Enzyme-Linked Immunosorbent Assay
WB	Western Blot
SA-β-Gal	Senescence-Associated β-Galactosidase
t-BHP	tert-Butyl hydroperoxide
KGN	Human granulosa-like tumor cell line
CCK-8	Cell Counting Kit-8
ROS	Reactive Oxygen Species
SOD	superoxide dismutase
CAT	catalase
GPx	glutathione peroxidase
DCFH-DA	2′,7′-dichlorodihydrofluorescein diacetate
D	Gestation Day
JAK1	Janus Kinase 1
STAT3	Signal Transducer and Activator of Transcription 3
RT-qPCR	reverse-transcription quantitative PCR
PPI	protein–protein interaction
KEGG	Kyoto Encyclopedia of Genes and Genomes.
GO	Gene Ontology
